# The Dual-Responsive Interaction of Particulated Hyaline Cartilage and Plasma Rich in Growth Factors (PRGF) in the Repair of Cartilage Defects: An In Vitro Study

**DOI:** 10.3390/ijms241411581

**Published:** 2023-07-18

**Authors:** Eduardo Anitua, Roberto Prado, Jorge Guadilla, Mohammad H. Alkhraisat, Patricia Laiz, Sabino Padilla, Montserrat García-Balletbó, Ramón Cugat

**Affiliations:** 1 Eduardo Anitua Foundation for Biomedical Research, 01007 Vitoria, Spain; roberto.prado@bti-implant.es (R.P.); mohammad.hamdan@bti-implant.es (M.H.A.); sabino.padilla1958@gmail.com (S.P.); 2 Regenerative Medicine Laboratory, BTI-Biotechnology Institute IMASD, 01007 Vitoria, Spain; 3 University Institute for Regenerative Medicine & Oral Implantology—UIRMI (UPV/EHU-Fundación Eduardo Anitua), 01007 Vitoria, Spain; 4 Osakidetza Basque Health Service, Araba University Hospital, 01009 Vitoria, Spain; jorge.guadilla@ucatrauma.com; 5 Arthroscopic Surgery Unit, Hospital Vithas Vitoria, 01008 Vitoria, Spain; 6 Department of Surgery and Radiology and Physical Medicine, Faculty of Medicine and Nursing, University of the Basque Country (UPV/EHU), 01006 Vitoria, Spain; 7 Fundación García Cugat para Investigación Biomédica, 08023 Barcelona, Spain; plaiz@institutocugat.com (P.L.); montse.garcia@institutocugat.com (M.G.-B.); ramon.cugat@sportrauma.com (R.C.); 8 Instituto Cugat, Hospital Quirónsalud, 08023 Barcelona, Spain; 9 Mutualidad de Futbolistas Españoles, Delegación Catalana, 08010 Barcelona, Spain

**Keywords:** chondral defects, particulated cartilage, PACI, platelet-rich plasma, PRGF, chondrocyte, growth factors, fibrin

## Abstract

The treatment of chondral and osteochondral defects is challenging. These types of lesions are painful and progress to osteoarthritis over time. Tissue engineering offers tools to address this unmet medical need. The use of an autologous cartilage construct consisting of hyaline cartilage chips embedded in plasma rich in growth factors (PRGF) has been proposed as a therapeutic alternative. The purpose of this study was to dig into the potential mechanisms behind the in vitro remodelling process that might explain the clinical success of this technique and facilitate its optimisation. Chondrocyte viability and cellular behaviour over eight weeks of in vitro culture, type II collagen synthesis, the dual delivery of growth factors by hyaline cartilage and PRGF matrix, and the ultrastructure of the construct and its remodelling were characterised. The main finding of this research is that the cartilage fragments embedded in the three-dimensional PRGF scaffold contain viable chondrocytes that are able to migrate into the fibrin network, proliferate and synthesise extracellular matrix after the second week of in vitro culture. The characterization of this three-dimensional matrix is key to unravelling the molecular kinetics responsible for its efficacy.

## 1. Introduction

Adult chondral and osteochondral lesions [1] are painful and evolve through a low-grade inflammatory process towards an osteoarthritic joint [2,3,4]. As a challenging endeavour, intense efforts have been invested in the engineering of autologous biomaterials for the repair and regeneration of articular cartilage defects [5,6,7]; however, satisfactory outcomes remain elusive [5,6,7,8]. The pivotal driver in cartilage homeostasis behind any attempts at cartilage restoration is the chondrocyte, which is a post-mitotic cell that requires a pericellular microenvironment with optimal biological and mechanical properties. Under these conditions, chondrocytes will survive, proliferate, migrate, and secrete extracellular matrix (ECM) to maintain the homeostasis of the cartilage [8,9]. Several strategies to repair injured cartilage have been proposed to create a good chondrocyte microenvironment. Autologous chondrocyte implantation (ACI) and particulated cartilage auto/allograft strategies have shown positive mid-term clinical outcomes [8,10,11,12,13,14].

Articular cartilage is an avascular and hydrated tissue with functional properties that transfer, partially absorb, and dissipate mechanical forces, which render the synovial joints’ movements frictionless and pain free [15]. Its unique and well-stratified chondrocytes and dense ECM allocation (devoid of nerves and vessels) generate a durable tissue that is highly resistant to complex loading patterns. However, the price to pay is the lack of healing capacity following mechanical and chemical insults or trauma-related injuries [1].

Over the last decade, different types of cartilage constructs have been described to induce cartilage healing [6,8,16,17]. All the cartilage constructs have been based on particulated cartilage auto/allografts that have been mixed with different agents like fibrin glue, collagen, and platelet-rich plasma (PRP). These constructs have exhibited variable degrees of experimental and clinical efficacy [6,8,16,17]. Among these biomatrices, our group proposed and designed an autologous cartilage construct consisting of hyaline cartilage chips embedded in plasma rich in growth factors (PRGF) [12,13,16,17,18]. Experimental studies in sheep have shown the capacity of this scaffold to restore the chondral lesion with hyaline cartilage with a nearly normal macroscopic ICRS assessment [17,18]. Clinical studies in young active individuals (full-thickness cartilage or osteochondral defects) have also demonstrated the advantage of our approach in providing long-term pain relief and high functional and MRI-based outcomes [16,17]. Moreover, the technique is a single-step, safe, and cost-effective surgical procedure (it does not require fixation techniques). The freshly activated autologous PRGF matrix robustly adhered to and filled the cartilage defect bed [13,16] due to its viscoelastic and adherent attributes [19]. However, its restorative potential strongly relies on chondrocyte behaviours and synthetic activities.

Thus, there is a need to understand the mechanisms through which this matrix could affect the healing of cartilage defects [13,16]. In this in vitro study, several relevant biological aspects have been assessed [20,21,22]. Chondrocyte viability and their cellular behaviour in the matrix have been monitored for eight weeks. Emphasis has also been placed on the assessment of the dual release of growth factors by the hyaline cartilage and the PRGF matrix. Moreover, structural characterization of the construct and its remodelling have been performed. Therefore, the purpose of this study was to dig into the potential mechanisms behind the in vitro remodelling process that might explain the clinical success of this technique and facilitate its optimisation.

## 2. Results

### 2.1. Characterization of PRGF and Cartilage Particles

The non-activated liquid PRGF was characterised using haematological analysis. The PRGF was found to be enriched in platelets (a two-fold increase in comparison with the level in the peripheral blood) and almost devoid of leukocytes and erythrocytes (Table 1). PRGF was categorised as pure-PRP (P-PRP), as it contains neither leukocytes nor erythrocytes [23] and is coded as 24-00-11 [24].

**Figure 1:**
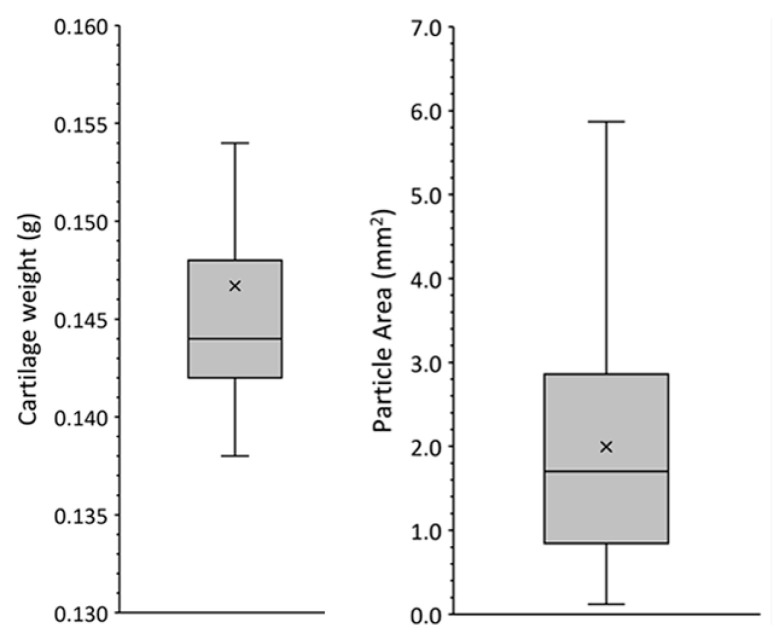
Distribution of median weight and size of cartilage particles. The graphs show the median, mean, and interquartile range of the mean weight of cartilage fragments mixed with PRGF in the different experiments, and the mean size of these fragments. *n* = 66 wells for weight determination; *n* = 121 fragments for area calculation.

After fragmentation and prior to mixing with PRGF, the cartilage was distributed among individual Petri dishes for the different experiments. The mean weight per dish was 0.147 ± 0.007 g, with a median of 0.144 (out of 66 prepared wells) (Figure 1F). Image analysis was utilised to quantify the size of the cartilage fragments used in the various experiments. The mean size was 2.0 ± 1.4 mm^2^, with a median of 1.7 (out of 121 fragments) (Figure 1).

### 2.2. Cell Culture, Histology, and Immunohistochemistry

**Figure 2:**
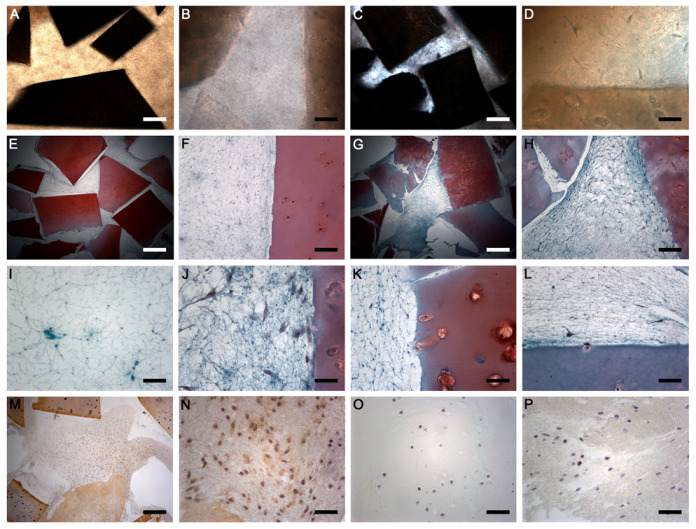
Cell culture images (**A**–**D**) showing the three-dimensional matrix at time zero (**A**,**B**) and after four weeks of in vitro culture (**C**,**D**). Safranin O staining (**E**–**L**) showing representative images at t0 (**E**,**F**) and t4 (**G**,**H**). Detail at ×40 magnification of the matrices at t4 (**I**–**L**). Immunohistochemistry of collagen type II (**M**,**N**) showing deposition of collagen II between fibrin fibres. Ki-67 immunohistochemistry (**O**,**P**) marking the nuclei of the proliferating cells. Original magnifications: ×2.5 (**A**,**C**,**E**,**G**); ×10 (**F**,**H**); ×40 (**B**,**D**,**I**–**P**). Scale bars: (**A**,**C**,**E**,**G**) 800 µm; (**F**,**H**) 200 µm; (**B**,**D**,**I**–**P**) 50 µm.

The constructs of particulated cartilage and PRGF were stable for eight weeks, although some thinning of the fibrin component was observed. However, the matrices formed of only PRGF (without cartilage fragments) were progressively dissolved after 3–4 weeks of culturing. By the fifth week, no PRGF matrix remained. Cell cultures were observed for at least four weeks for the constructs of particulate cartilage and PRGF. Freshly prepared samples (time 0) exhibited cartilage fragments dispersed within the fibrin network (Figure 2A). No cells were observed within the fibrin network of homogeneous appearance (Figure 2B). In general, the presence of cells was observed near the cartilage fragments after the second week of culturing. After four weeks, this presence was marked, with cells being distributed three-dimensionally (Figure 2C) within the fibrin network between the cartilage fragments (Figure 2D). These direct observations were confirmed by the histological analysis. At time 0, cartilage fragments showed variable Safranin O staining and a normal appearance and were distributed throughout the fibrin network (Figure 2E). In more detail, a cell-free fibrin network was observed in close contact with the cartilage (Figure 2F). After 4 weeks, the fibrin network was still present, although the remodelling process had begun (Figure 2G). Scattered chondrocytes of variable morphologies were observed within the PRGF network (Figure 2H). Platelet aggregates of different sizes could be observed scattered between the fibrin fibres (Figure 2I). The cells that had emerged from the cartilage fragments showed variable morphologies as they were embedded within the three-dimensional fibrin network (Figure 2J). The fragmentation of the cartilage during preparation increased the contact area between the cartilage and fibrin, which allowed for cell outgrowth. In several cases, empty lacunae could be observed close to the edge (Figure 2K) or with the chondrocyte itself still inside the lacuna (Figure 2L).

The chondrocyte outgrowth from cartilage fragments was assessed according to the score of Levinson et al. It was noted that the cells began to outgrow the cartilage fragments after two weeks of incubation. This outgrowth was initially focal, with cells mainly observed on or near the cartilage fragments (score 1). Later, the cells began to migrate and proliferate into the PRGF-fibrin network (score 2). This situation was observed between weeks 3 and 4. The scores obtained for the four samples analysed during the four weeks were as follows: 0 (week 1); 0.8 ± 0.5 (week 2); 1.3 ± 1.0 (week 3); and 2.0 ± 0.0 (week 4).

It should be noted that positive type II collagen cells were found in the fibrin network, which suggested that the chondroid phenotype was retained (Figure 2M). Further, the deposition of type II collagen in the fibrin network indicated the remodelling of the fibrin into the extracellular matrix of hyaline cartilage (Figure 2N). Cell proliferation was also measured by immunohistochemistry for the Ki-67 protein, which is only present when cells have entered the cell cycle. Positive staining was not observed in the chondrocytes within the cartilage fragments (Figure 2O) but was observed in cells that had outgrown the fragments and were scattered within the PRGF network (Figure 2P).

### 2.3. Hoechst 33342 and Live/Dead Staining

**Figure 3:**
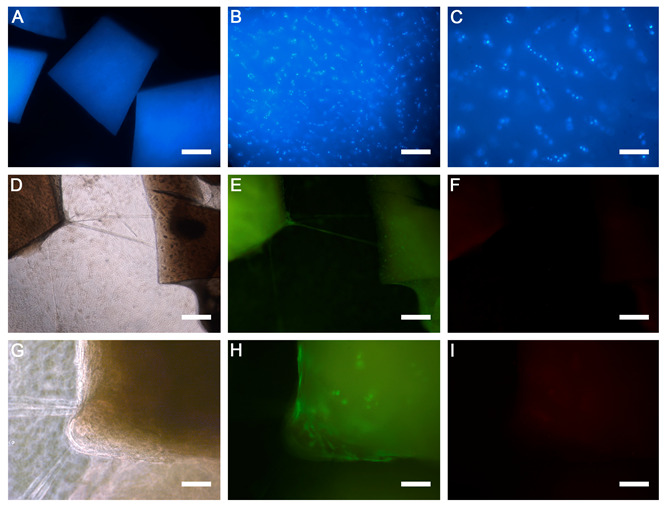
Microphotographs illustrating (**A**) the background staining of the cartilage fragments with Hoechst 33,342 at low magnification. (**B**) At higher magnification, the nuclei of the chondrocytes stained with Hoechst 33,342 can be seen dispersed within a fragment of cartilage. (**C**) Detail of the chondrocytes at higher magnification (Hoechst 33,342). Sequence of microphotographs from two different fields, at low (**D**,**E**) and high magnification (**F**–**I**), showing the viability of chondrocytes in a sample of 4 weeks in culture. The phase-contrast images (**D**,**G**) show the cartilage fragments. In the fluorescence channels, the green colour shows live cells (**E**,**H**), while no dead cells were visible in the red channel (**F**,**I**). Original magnifications: ×2.5 (**A**); ×5 (**D**–**F**); ×20 (**B**,**C**,**G**–**I**). Scale bars: (**A**) 800 µm; (**D**–**F**) 400 µm; (**B**,**C**,**G**–**I**) 100 µm.

Under low magnification, the cartilage fragments could be seen to have some background in the Hoechst 33342 staining (Figure 3A). However, at higher magnification, chondrocyte nuclei were clearly visible within the cartilage matrix (Figure 3B,C). The viability of the chondrocytes within the cartilage fragments and those cells that had migrated beyond the PRGF matrix was observed for four weeks via live/dead staining. It was found that almost all cells were stained green with calcein-AM (alive cells that did not lose membrane integrity), both within and outside the cartilage fragments. Some dead cells stained red (ethidium homodimer-1) were observed very sporadically. Representative images of samples after four weeks of culturing are shown (Figure 3D,I). Phase-contrast images clearly demonstrated the cartilage fragments (Figure 3D,G). The images in the green channel indicated live cells (Figure 3E,H), while no dead cells were visible in the red channel (Figure 3F,I).

### 2.4. Ultrastructural Analysis

**Figure 4:**
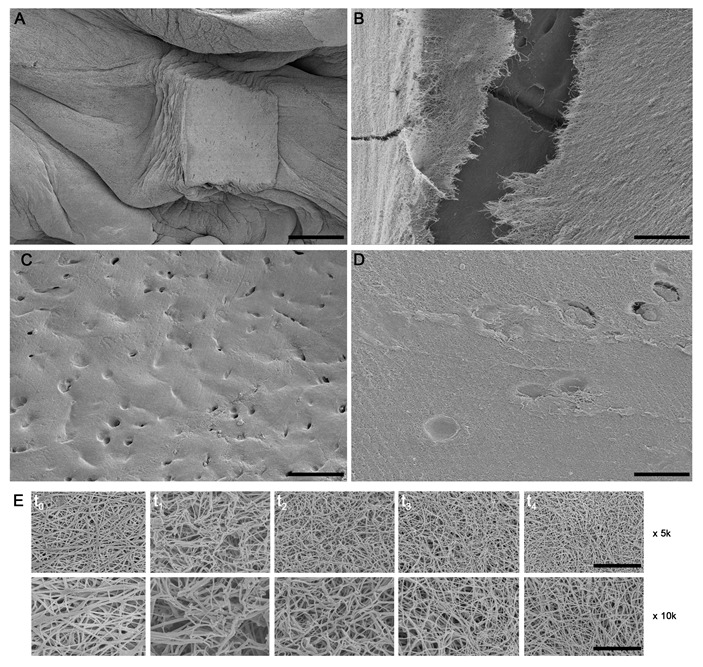
Scanning electron microscopy (SEM) images showing (**A**) cartilage fragments uniformly embedded within the fibrin network of PRGF. (**B**) Photomicrograph showing an area where the fibrin matrix has broken down and cartilage fragments are visible. (**C**) Chondrocyte lacunae exposed on the surface of a cartilage fragment. (**D**) High magnification image showing several empty lacunae and other lacunae with chondrocytes inside. (**E**) PRGF fibrin network over 4 weeks of incubation (t_0_–t_4_) showing that its three-dimensional structure has been retained. Original magnifications: ×50 (**A**); ×200 (**C**); ×500 (**B**); ×1000 (**D**); ×5000 (**top row of E**); ×10000 (**bottom row of E**). Scale bars: (**A**) 500 µm; (**B**) 50 µm; (**C**) 125 µm; (**D**) 25 µm; (**E**) top line 10 µm and bottom line 5 µm.

SEM images revealed that the cartilage fragments were uniformly embedded within the PRGF fibrin network (Figure 4A). In some areas where the three-dimensional PRGF matrix was ruptured, cartilage fragments and even lacunae could be observed (Figure 4B). The density of chondrocyte lacunae that were exposed on the surface varied according to the zone of cartilage fragment origin (Figure 4C). Some chondrocytes were even observed in their lacunae (Figure 4D). Conversely, it was observed that the three-dimensional structure of the PRGF fibrin network was preserved over the four weeks (Figure 4E).

**Figure 5:**
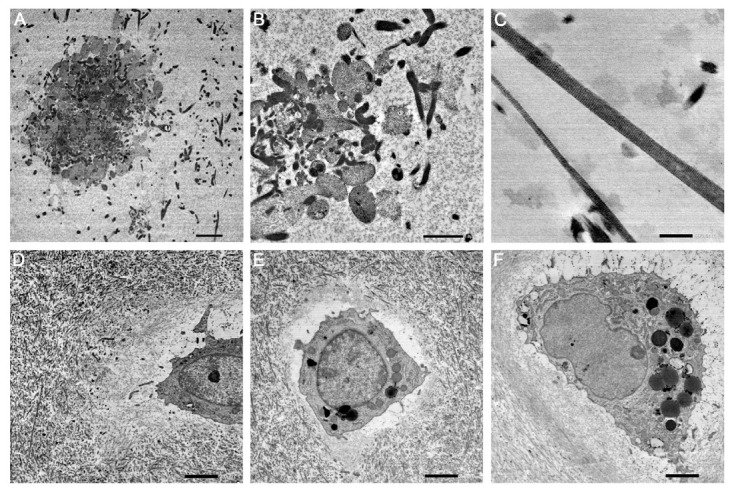
Transmission electron microscopy (TEM) images showing (**A**) platelet aggregates scattered throughout the three-dimensional PRGF matrix after formation and (**B**) after three weeks of culture. (**C**) Typical striation of the fibrin fibres after three weeks of in vitro culture. (**D**–**F**) Live chondroid cells within the cartilage fragments were observed during the whole incubation period. Scale bars: 500 nm (**C**); 1 µm (**B**); 2 µm (**A**,**D**–**F**).

As expected with the TEM technique, platelet aggregates were observed scattered throughout the three-dimensional PRGF matrix after formation (Figure 5A). These aggregates were preserved over time (e.g., three weeks of culturing) (Figure 5B). The fibrin fibres were also conserved after this time and exhibited their typical striation (Figure 5C). Regarding the cellular components, chondroid cells were observed throughout the incubation period (Figure 5D–F). In contrast to the previously described histological sections, no scattered cells were found in the PRGF matrix. This might have been due to the TEM magnifications being much higher; thus, the probability of encountering cells was much lower.

### 2.5. Release Kinetics of Biomolecules

Samples were collected twice a week, and the release kinetics of the biomolecules were quantified by ELISA. Simultaneously, the conditioned medium of the PRGF matrices without cartilage fragments was also collected as a control. Significantly, in all cases, a more pronounced release was found during the first days of incubation, which gradually reached a plateau over 4.5 weeks. As a reference value, the concentrations of IGF-1, HDGF, and COMP in the PRGF supernatant were 38.43 ± 0.86 ng/mL, 993.56 ± 109.58 pg/mL, and 185.92 ± 10.43 ng/mL, respectively.

**Figure 6:**
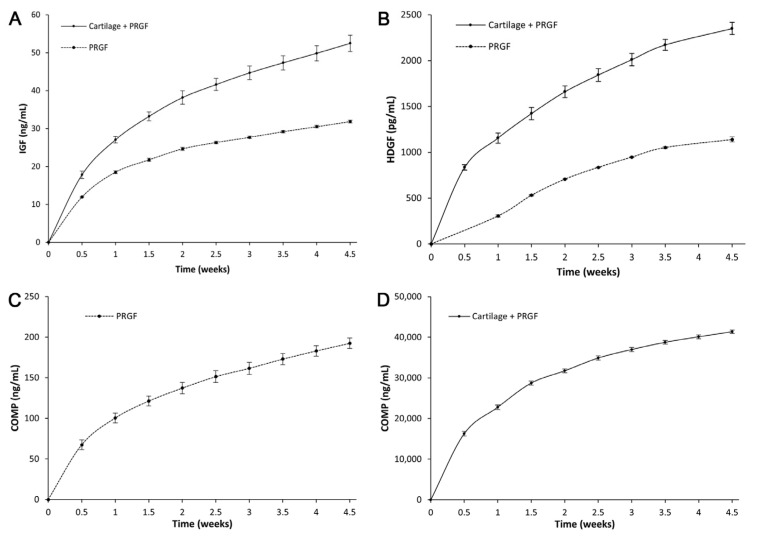
Release kinetics of growth factors from the constructs consisting of cartilage fragments and PRGF and PRGF matrix alone. (**A**) IGF-1, (**B**) HDGF, COMP release in (**C**) the PRGF matrix, and in (**D**) the constructs of cartilage and PRGF. The COMP release kinetics are shown in two separate graphs due to the different order of magnitudes.

Figure 6 shows the release kinetics of these growth factors from the PRGF matrix and the construct of cartilage and PRGF. In the case of IGF-1 (Figure 6A), a higher level of release was observed in the presence of cartilage fragments. A similar pattern was observed for the HDGF (Figure 6B), but it was more pronounced as the composed matrix released two to four times more HDGF than did the PRGF matrix. The COMP also showed a similar release pattern to the previous ones; however, within the PRGF matrices, the cumulative release concentration was very low and did not exceed 200 ng/mL (Figure 6C). The matrix consisting of cartilage and PRGF showed a high concentration, which was approximately two hundred times higher than the matrices comprised of PRGF alone, which clearly demonstrated the contribution of the cartilage fragments to COMP release (Figure 6D). The COMP release kinetics are shown in two separate graphs due to the different order of magnitudes, as discussed above.

## 3. Discussion

The successful restoration of articular cartilage in chondral and osteochondral lesions is a dynamic, multifactorial, and complex process where pivotal players are the type of biological construct carrying viable chondrocytes and the microenvironment where the biological construct is implanted. This in vitro research presents evidence that a biological construct comprised of cartilage fragments embedded within a three-dimensional PRGF scaffold contained viable chondrocytes, which had the capacity to migrate within the fibrin network, proliferate, and synthesise extracellular matrix after two weeks of in vitro culturing. Several in vivo experimental and clinical studies have reported that the regenerative approach investigated in this in vitro research promoted the synthesis of collagen type II [17,18], provided clinical long-term pain relief, and had high functional and MRI-based outcomes [12,16].

Cartilage fragmentation was performed mechanically using a scalpel blade, which generated an inherent size variability similar to that reported by Levinson et al. in 2019 [20] (2.02 ± 0.90 mm^2^ compared to 2.0 ± 1.4 mm^2^ in this study). As recently stated by Evuarherhe et al. [25], it is complex to reliably measure the weight of cartilage fragments due to their sticky and fragile nature. For this study, all samples were weighed, and dryness was always avoided; most of the aqueous component was removed without compromising the viability of the samples.

Our results are consistent with recent research that observed cell outgrowth from cartilage fragments cultured in various matrices [20,26,27]. However, this is in contrast to the work of Andjelkov et al. [28], which did not observe any cell outgrowth. This might have been due to the inclusion of cartilage fragments from a commercial matrix that contained high fibrinogen and thrombin concentrations, which would generate a three-dimensional network that was too compact with a very small pore size.

The presence of anabolic, trophic, and mitogenic growth factors within the fibrin matrix likely avoided the cell’s programmed death by providing microenvironmental signals in the form of TGF-β, IGF-1, PDGF, and FGF, among others [29]. In this study, the release kinetics of several molecules involved in cartilage regeneration, including growth factors, were determined, and IGF-1 (the most abundant GF present within PRGF [30]) was a major anabolic signal for cartilage growth and homeostasis. The IGF-1 signalling pathway has been implicated in three pivotal processes during chondrogenesis: (a) modulation of anabolism through chondrocyte proliferation, stimulation of ECM synthesis, and antiapoptotic effects [31,32,33], (b) modulation of mechanosensitivity of chondrocytes as the initially required trophic signal [34], and (c) protective and anti-inflammatory effects through the inhibition of the NFkB signalling pathway in chondrocytes [33,35]. The IGF-1 concentration has been higher in the constructs of cartilage and PRGF than in the PRGF matrix, which may indicate an additional contribution from the cartilage fragments. The HDGF factor is related to chondrocyte survival and the repair of cartilage injuries [36]. This is the first time that its presence in PRP has been characterised. The presence of HDGF in cartilage samples was recently described [37,38]. These authors hypothesised that HDGF was a heparan sulphate-binding growth factor released from the pericellular cartilage matrix in response to mechanical damage through a sodium-dependent mechanism. The exact role of HDGF in cartilage regeneration and the significance of its presence in PRGF are yet to be elucidated. The more pronounced presence of HDGF in the cartilage/PRGF construct suggested that cartilage ECM possessed this compound and might contribute to the binding of several GFs stemming from PRGF. COMP is a member of the extracellular matrix of cartilage and promotes the secretion and assembly of collagen while providing stability to the extracellular matrix [39]. Further, it has recently been described that COMP not only binds to different ECM components but also interacts with growth factors to provide a reservoir for their uptake by cells [40]. Among the myriad roles of this molecule, COMP has also been reported to be a potent inhibitor of chondrocyte apoptosis through the blockade of caspase-3 activation [41]. It may also be a chemotactic molecule for chondrocytes, thus contributing to the repopulation of damaged cartilage sites [42]. As noted, COMP release in the cartilage matrices was approximately 200 orders of magnitude higher than that within the PRGF matrices, which explains why they were plotted in individual graphs. The dual participation of the cartilage matrix and PRGF matrix to provide signalling molecules, growth factors, and cytokines should be considered when assessing the healing properties of the cartilage fragments/PRGF construct. It is arguable that the presence of fibrin would also modulate the release kinetics of these biomolecules and properly enhance their availability.

Moreover, several soluble biomolecules embedded within the fibrin network (e.g., PDGF, SDF-1, TGF-β, CCL5, and fibronectin, among others) have been shown to be involved in cell recruitment and homing. PRP could favour the chondrogenic differentiation of chondroprogenitors or MSCs from subchondral mesenchymal progenitor cells [33,43,44,45]. In addition, PRGF may have dampened the detrimental chondrocyte response to the mechanical stress during shaving and mechanical mincing and the subsequent activation of pro-inflammatory gene expression through the NFkB signalling pathway [46,47,48]. The rapid integration of minced cartilage into the cell-compatible scaffold proceeded as the PRGF liquid/gel matrix sought to minimise the mechanical and chemical stresses experienced via the excision and mincing processes, thereby promoting an anti-inflammatory and survival milieu [30].

Since chondrocytes are known to be highly dependent on their microenvironment, one advantage of the technique proposed here is that chondrocytes within cartilage fragments can remain viable with a conserved phenotype. Conversely, those cells involved in outgrowth from the cartilage fragments may lose their chondroid phenotype. However, we observed that type II collagen synthesis can be maintained in vitro. Recently, Elson et al. [49] demonstrated that most chondrocytes in osteochondral organ culture can remain viable for up to 20 days. Our results are consistent with this study, as virtually all cells, both within and outside the cartilage fragments, were found to be viable (as measured by live/dead staining) during the four weeks of culturing.

Overall, the results obtained in this and preceding studies allowed us to postulate an in vivo regeneration mechanism that supported the use of cartilage fragments embedded in a three-dimensional PRGF matrix. Our hypothesis of the in vivo regeneration mechanism involves a dual approach for cell recruitment. On the one hand, mesenchymal cells from the subchondral bone are recruited, while on the other hand, chondrocytes from both the cartilage fragments and the edges of the lesion are attracted by the growth factors that are deposited within the PRGF matrix. The growth factors [50] retained in the fibrin by the heparin-binding domain (FGF-b, HGF, PDGF-AB/BB, IGF-1, TGF-β1, etc.) serve as attracting factors for MSCs and contribute to their differentiation into chondrocytes.

However, this study has several limitations, the first of which is that this is an in vitro study for which the tissue (the cartilage fragments) was derived from the patient, while the PRGF was obtained from donor blood. In the clinic, the technique was completely autologous; thus, it will be a matter for further investigation to determine whether it is of relevance that the tissue donors are distinct from the blood donors. Secondly, only a small number of samples were used, which were obtained from five patients who underwent knee replacement. However, not all could be employed for all tests, which was due in part to the high contamination rate of the in vitro cultures derived from the cartilage fragments. A third limitation is that no comparative study was conducted with other commercially available systems (e.g., juvenile cartilage systems).

## 4. Materials and Methods

### 4.1. Study Design and IRB Approval

The study protocol (code EndoCarTech, record 2021-007) was approved on 19 February 2021 by the Institutional Review Board of the CEI OSI ARABA in accordance with the international ethical standards of the revised World Medical Association Declaration of Helsinki, amended in 2013 in Brazil. Patients and blood donors were informed about this study and provided written informed consent.

### 4.2. Cartilage

#### 4.2.1. Human Tissue Collection

Osteochondral tissues that are normally discarded following total knee arthroplasty (TKA) were obtained from five patients (three women and two men, aged 73.3 ± 2.9 years) through the Basque Biobank. Briefly, tissue fragments were collected from the femoral condyle in the operating room and submerged in sterile phosphate-buffered saline (PBS) supplemented with amphotericin B (2.5 µg/mL) and gentamicin (50 µg/mL). The tissue fragments were transferred to the laboratory at 4 °C in less than 2 h and processed immediately.

#### 4.2.2. Preparation of Particulated Cartilage

**Figure 7:**
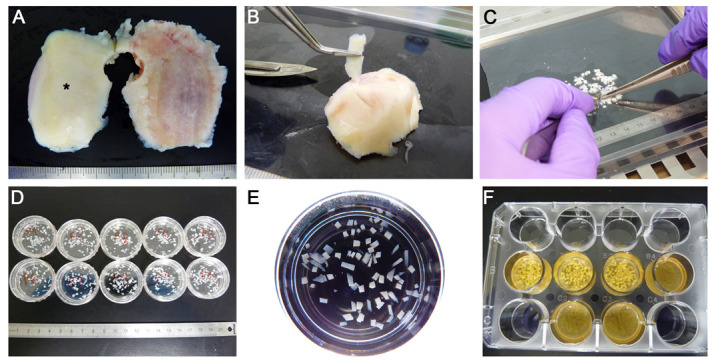
Detail of sample collection and preparation. (**A**) Cartilage samples were examined, and fragments of normal macroscopic appearance (*) were selected. (**B**) The cartilage was cut into slices, avoiding the subchondral bone. (**C**) These slices were then mechanically minced into smaller fragments using a scalpel, similar to those used in the clinical setting. (**D**) Cartilage fragments were kept submerged and homogeneously distributed in Petri dishes. (**E**) Each dish was photographed independently to obtain the size distribution of the cartilage fragments. (**F**) Finally, three-dimensional matrices of PRGF and PRGF-embedded cartilage fragments were obtained.

Upon arrival, samples were washed twice in sterile PBS supplemented with amphotericin B and gentamicin. The fragments showing macroscopic signs of osteoarthritis were discarded, and experiments were performed only on the cartilage of normal macroscopic appearance (Figure 7A) [51]. First, the cartilage was sliced, avoiding the subchondral bone (Figure 7B). These slices were then mechanically minced into smaller fragments with the aid of a scalpel (blade handle #4 and blade #23), similar to the ones used in the clinical setting [13], approximately 1–3 mm^2^ (Figure 7C). Care was taken to ensure that the fragments did not dry out.

#### 4.2.3. Cartilage Particle Size Distribution

The cartilage fragments were kept submerged in sterile PBS supplemented with amphotericin B and gentamicin and homogeneously distributed in Petri dishes (35 mm diameter), which were placed on a black background underneath (Figure 7D). Photographs were obtained using a stereomicroscope MZ6 (Leica Microsystems, Wetzlar, Germany) coupled with a DFC 300 FX digital camera (Leica Microsystems) (Figure 7E), which were then analysed using the ImageJ software (version 1.53, National Institutes of Health, Bethesda, MD, USA).

### 4.3. PRGF Preparation and Characterization

#### 4.3.1. PRGF Preparation

PRGF was prepared according to Anitua’s protocol [52,53]. Briefly, blood (145–170 mL) from five healthy donors (three women and two men, aged 44.6 ± 9.5 years) was withdrawn into 9-mL tubes containing 0.4 mL of 3.8% (*w/v*) sodium citrate as an anticoagulant (EDK2, BTI Biotechnology Institute, S.L., Vitoria, Spain). The tubes were then immediately centrifuged at 580 *g* for 8 min at room temperature using a System V centrifuge (BTI Biotechnology Institute, S.L., Vitoria, Spain). After centrifugation, platelets were distributed in the plasma column in a concentration gradient, lower at the top of the column and higher near the leukocyte layer. To obtain non-activated liquid PRGF, the plasma column was separated into two fractions: The F1 fraction, the upper 50% of the column, with a lower platelet concentration, and the F2 fraction (50% of the lower volume of the column) with a higher platelet concentration. The buffy coat was excluded. Equal volumes of each fraction were pooled. This PRGF formulation was employed to create the constructs that included the cartilage fragments and to produce the PRGF supernatant for cell culture.

A portion of the PRGF was activated to obtain a PRGF supernatant for use as a supplement to the cell culture medium. The PRGF was activated with 10% CaCl_2_ (PRGF activator, BTI Biotechnology Institute, S.L., Vitoria, Spain) at a proportion of 20 µL/mL. After one hour of incubation at 37 °C, the formed clot was discarded, and the PRGF supernatant was collected, filtered with 0.22 μm filters, aliquoted, and stored at −80 °C until use.

#### 4.3.2. PRGF Characterization and Classification

A complete count with a five-part differential (Pentra ES 60, Horiba ABX SAS, Montpelier, France) was performed on whole blood and non-activated liquid PRGF. Further, the platelet yield (%) and the platelet concentration factor relative to the level of peripheral blood were calculated. PRGF was classified according to the obtained data.

### 4.4. Preparation of Particulated Cartilage and PRGF Matrix

The preparation of the three-dimensional matrix proceeded by mixing the freshly activated PRGF with the cartilage fragments in 12-well plates (3.5 cm^2^/well). The particles placed in each well were weighed (0.140 g/well), although these values were only indicative due to the aim of avoiding the complete drying of the cartilage fragments. Next, 1 mL of PRGF was activated with 20 µL of CaCl_2_ and was immediately added to each well. The constructs were allowed to coagulate in the incubator at 37 °C for 30 min (Figure 7F). Subsequently, the appropriate culture medium was added. As a control, PRGF matrices without cartilage were prepared.

### 4.5. Cell Culture

The samples were cultured in 12-well cell culture plates with 540 μL of DMEM/F-12 plus 2 mM glutamine and 50 µg/mL gentamicin), supplemented with 60 μL of PRGF supernatant, and maintained at 37 °C in a humidified atmosphere with 5% CO_2_ for 8 weeks. The culture medium was changed bi-weekly.

### 4.6. Histology and Immunohistochemistry

Briefly, the samples collected at weekly intervals were fixed in 10% (*v*/*v*) neutral buffered formalin for 24 h, dehydrated in a graded series of alcohols, paraffin-embedded, and sectioned at 5 μm thickness. The obtained sections were stained with hematoxylin and eosin and Safranin O. The synthesis and deposition of collagen II in the constructs were determined by immunohistochemistry (Abcam, Cambridge, UK), while the proliferating cells were detected by staining with anti-Ki-67 (Abcam, Cambridge, UK). All sections were examined via conventional optical microscopy using a Leica DMLB microscope (Leica Microsystems, Wetzlar, Germany) and photographed with a Leica DFC 300 FX digital camera (Leica Microsystems). The micrographs were analysed using the ImageJ software (version 1.53, National Institutes of Health, Bethesda, MD, USA).

The chondrocyte outgrowth was evaluated following the score developed by Levinson et al. [20]. Briefly, on a scale of zero to two, zero referred to no cell migration from the cartilage fragments to the three-dimensional fibrin matrix, while one point referred to very limited cell migration with one or two exit sites, and two referred to samples that contained cells that were scattered throughout the fibrin network.

### 4.7. Immunofluorescence

#### 4.7.1. Hoechst 33342

After discarding the culture medium, the matrices were washed twice with PBS and stained with Hoechst 33342 (Molecular Probes, Thermo Fisher Scientific, Waltham, MA, USA) at 6.1 µM for 20 min at RT in the dark. Afterwards, any excess dye was removed, and microphotographs were taken.

#### 4.7.2. Live/Dead Staining

To investigate the viability of chondrocytes, both within the cartilage fragments and those that have migrated into the three-dimensional fibrin matrix, a live/dead assay was performed using a LIVE/DEAD cell imaging kit (Molecular Probes, Thermo Fisher Scientific, Waltham, MA, USA), according to the manufacturer’s instructions. Briefly, after culturing, the cell culture medium was discarded and incubated with the kit mixture. This solution contained green fluorescent calcein-AM to indicate intracellular esterase activity and red fluorescent ethidium homodimer-1 to indicate the loss of plasma membrane integrity. Next, it was incubated for 30 min at RT in the dark. Following incubation, representative images were obtained from different areas using a wide-field fluorescent microscope, which highlighted live cells as green and dead cells as red.

### 4.8. Ultrastructural Analysis

Scanning (SEM) and transmission (TEM) electron microscopy techniques were employed for the ultrastructural analysis of particulated cartilage embedded in PRGF. Briefly, the samples collected at one-week intervals were rinsed with PBS, fixed with 2% glutaraldehyde in a 0.1 M cacodylate buffer for 4 h and washed 3 times in a cacodylate-sucrose buffer (0.1 M cacodylate, 6.5% sucrose, pH 7.4). Subsequently, the samples were post-fixed with osmium tetroxide (1% OsO_4_ in 0.1 M cacodylate) for 1 h at 4 °C in the dark and washed in 0.1 M cacodylate. Finally, the samples were dehydrated using increasing ethanol concentrations (30, 50, 70, 96, and 100%). At this point, the samples were separated for each of the two imaging techniques (SEM and TEM) and processed independently. For SEM, the samples were incubated twice in hexamethyldisilazane for 10 min and allowed to dry prior to examination with a Hitachi S-4800 electron microscope. For TEM imaging, the samples were incubated in propylene oxide for 1 h, then in increasing concentrations of Epon resin, and finally embedded in Epon. Ultrathin sections were stained with uranyl acetate and lead citrate and examined using a Philips EM208S electron microscope.

### 4.9. Assessment of Biomolecule Release Kinetics

The characterisation of the bioactive release of different molecules over time from the PRGF/cartilage constructs and from the PRGF alone was performed. Samples were maintained in a cell culture incubator at 37 °C, 5% CO_2_, and 95% humidity. The culture medium was changed and collected every 2–3 days for at least four weeks. After each interval, the incubation medium was immediately centrifuged at 400× *g* for 10 min at room temperature, after which the supernatant was distributed into aliquots and stored at −80 °C until use. The quantification of insulin-like growth factor 1 (IGF-1), cartilage oligomeric matrix protein (COMP), and hepatoma-derived growth factor (HDGF) was performed in duplicate using enzyme-linked immunosorbent assay (ELISA) kits following the manufacturer’s instructions (R&D Systems, Inc. (Minneapolis, MN, USA) for IGF and COMP, and Fine Biotech Co. (Wuhan, China) for HDGF). The assay ranges were 0.1–4 ng/mL for IGF-1, 0.2–10 ng/mL for COMP, and 31.2–2000 pg/mL for HDGF. Changes in the absorbance were measured using a multimode microplate reader (Synergy H1, Agilent BioTek, Santa Clara, CA, USA), and the concentrations of the different molecules were calculated using Gen5 software, version 3.08 (Agilent BioTek, Santa Clara, CA, USA).

## 5. Conclusions

In conclusion, the particulated cartilage fragments embedded in a three-dimensional PRGF scaffold contained viable chondrocytes with the capacity to migrate into the fibrin network, proliferate, and synthesise extracellular matrix after two weeks of in vitro culturing. The characterization of this three-dimensional matrix is key to unravelling the molecular kinetics responsible for its efficacy and optimising the treatment for patients with chondral defects.

## Figures and Tables

**Table 1:** Characterisation of whole blood and PRGF from the donors (*n* = 5) used in the experiments. In all cases, a complete blood count with 5-part differential was performed. Leukocyte, erythrocyte, and platelet concentration factors with respect to peripheral blood level and platelet yield (%) are also shown. Data are expressed as mean ± SD; n.d., not detected.

	Whole Blood	PRGF
Leukocytes (×10^3^/μL)	6.89 ± 0.62	0.17 ± 0.10
Lymphocytes (%)	26.9 ± 8.8	n.d.
Monocytes (%)	5.0 ± 1.9	n.d.
Neutrophils (%)	63.3 ± 10.1	n.d.
Eosinophils (%)	3.9 ± 2.4	n.d.
Basophils (%)	0.9 ± 0.3	n.d.
Erythrocytes (×10^6^/μL)	4.65 ± 0.25	0.01 ± 0.01
Platelets (×10^3^/μL)	241 ± 42	476 ± 99
Mean platelet volume (fL)	7.5 ± 0.7	7.2 ± 0.7
Leukocyte concentration factor	1	0.02 ± 0.01
Erythrocyte concentration factor	1	0
Platelet concentration factor	1	2.0 ± 0.3
Platelet yield (%)	100	66.6 ± 15.6

## Data Availability

All the obtained data used to support the findings of this study are available from the corresponding author upon reasonable request.
